# Effectiveness of platelet-rich fibrin concentrates in alveolar ridge preservation after tooth extraction: A systematic review of randomized clinical trials with tomographic evaluation

**DOI:** 10.1016/j.clinsp.2026.101172

**Published:** 2026-09-30

**Authors:** Daniela Meneses-Santos, Anderson Carlos Marçal, Caio Melo Mesquita, Walbert de Andrade Vieira, Marcelo Dias Moreira de Assis Costa, João Botelho, Sigmar de Mello Rode, Luiz Renato Paranhos

**Affiliations:** a Department of Morphology, Center of Biological Sciences and Health, Universidade Federal do Sergipe (UFS), São Cristóvão, SE, Brazil; b Graduate Program in Dentistry, Universidade Federal de Uberlândia (UFU), Uberlândia, MG, Brazil; c Department of Dentistry, Endodontics Division, Centro Universitário das Faculdades Associadas de Ensino (UNIFAE), São João da Boa Vista, SP, Brazil; d Division of Dental Clinic, Oral Pathology and Surgery, School of Dentistry, Universidade Federal de Minas Gerais (UFMG), Belo Horizonte, MG, Brazil; e Egas Moniz Center for Interdisciplinary Research, Egas Moniz School of Health & Science, Almada, Portugal; f Department of Dental Materials and Prosthetics, Institute of Science and Technology, Universidade Estadual Paulista Julio de Mesquita Filho (UNESP), São José dos Campos, SP, Brazil; g Department of Orthodontics, School of Dentistry, Universidade Federal de Uberlândia (UFU), Uberlândia, MG, Brazil

**Keywords:** Alveolar ridge, Cone-beam computed tomography, Platelet-rich fibrin, Tooth extraction

## Abstract

**Purpose:**

To systematically review the scientific evidence on the effect of Platelet-Rich Fibrin (PRF) concentrates’ application on tomographically evaluated alveolar ridge preservation in post-extraction sockets.

**Methods:**

A systematic search was conducted in seven electronic databases, and two additional bases as sources of gray literature, with no language or date restrictions. Randomized clinical trials comparing the use of PRF concentrates with placebo or no intervention were included. Tooth extractions were performed atraumatically, and alveolar ridge preservation was assessed by cone-beam computed tomography, measuring ridge width (horizontal) and height (vertical) changes. Two reviewers assessed the risk of bias with RoB 2.0 tool. Meta-analysis and GRADE assessment were also performed.

**Results:**

A total of 2843 studies were initially identified. After duplicate removal and screening, 13 studies were included in the qualitative analysis, totaling 599 evaluated sockets. The included studies, published between 2016 and 2024, presented several methodological differences and reported controversial effects of PRF and L-PRF, compared to control groups. Individual risk of bias varied from high to some concerns mainly related to poor blinding and lack of reporting details. In meta-analysis, pooled estimates did not show statistical significance for either alveolar ridge width or height. Certainty of evidence was rated very low due to small sample size and wide confidence intervals crossing the null effect line.

**Conclusion:**

Based on very low evidence, the application of PRF concentrates did not demonstrate appreciable benefits or harms for alveolar ridge preservation. However, these findings should be interpreted with caution due to data heterogeneity and bias concerns in primary studies. Thus, the evidence is insufficient to recommend its sole use as a predictable ridge preservation technique, and there is a current need on well-designed and standardized randomized clinical trials in this research field.

**Registration:**

Protocol registered in the PROSPERO database (CRD42025638069).

## Highlights

• The sole use of PRF concentrates did not improve alveolar ridge preservation.
• Primary studies showed relevant heterogeneity in their methodology and outcomes.
• Bias concerns were mainly related to poor blinding and lack of reporting details.

## Introduction

Tooth extraction is one of the most frequently performed procedures in oral surgery, primarily indicated for dental caries (approximately 36% to 55%) and periodontal diseases (around 24.8% to 38%), followed by trauma and periapical lesions.^1^ After extraction, the alveolar remodeling process varies among individuals but is generally characterized by a gradual reduction in both the height and width of the alveolar bone. Histologically, healing begins with the formation of a blood clot, which matures into granulation tissue. This is followed by the development of a temporary matrix that is eventually replaced by immature bone. Over the course of several weeks, this initial bone formation undergoes progressive remodeling into lamellar bone and bone marrow.^2^, ^3^, ^4^

Bone augmentation procedures are often necessary for the treatment of atrophic alveolar ridges and may be performed either before or after dental implant placement. However, these approaches can increase patient morbidity, incur substantial financial costs, and prolong the overall treatment timeline.^5^ Alveolar preservation techniques comprise clinical strategies implemented at the time of tooth extraction to minimize postoperative bone resorption, thereby reducing the need for future reconstructive procedures. Despite their benefits, these techniques may require additional donor sites, increase surgical complexity, elevate costs, and extend the healing period.^6^

Autologous platelet-rich blood concentrates have been widely used in various fields of oral surgery and implantology as bioactive agents intended to promote tissue regeneration.^7^ Some beneficial effects of Platelet-Rich Fibrin (PRF) such as reduced postoperative discomfort and improved soft tissue healing are already described in the literature, particularly within the first 2- to 3-months after tooth extraction.^8^

PRF acts as a reservoir of key bioactive molecules, including Transforming Growth Factor β1 (TGF-β1), Vascular Endothelial Growth Factor (VEGF), Platelet-Derived Growth Factor (PDGF), and Insulin-like Growth Factors (IGFs).^9^ These growth factors are embedded within the fibrin matrix, allowing for their gradual and sustained release. Once released, they modulate the local microenvironment and facilitate tissue regeneration.^10^ In addition to growth factors, PRF can also release a variety of pro- and anti-inflammatory cytokines.^11^ Given these properties, platelet concentrates have been investigated as a promising alternative for alveolar ridge preservation.

Although prior evidence^8^ suggested potential benefits on alveolar ridge preservation, and several randomized clinical trials have examined the use of platelet concentrates for this purpose, the results remain inconclusive ‒ mainly due to methodological heterogeneity in application protocols, dimensional measurement techniques, and follow-up durations.^12^ To address this limitation, only studies utilizing Cone-Beam Computed Tomography (CBCT) were included in this review, as CBCT is recognized as a precise and reliable tool for assessing three-dimensional bone changes following tooth extraction and PRF interventions. This approach enables an objective evaluation of the biomaterials' effects on bone density and volume.^7^

In light of this context, the present systematic review aimed to gather and critically evaluate the available evidence, focusing on studies that employed tomographic assessment, in order to establish consistent criteria to guide clinical decision-making in the management of post-extraction sockets.

## Material and methods

### Protocol registration

The protocol was reported according to the Preferred Reporting Items for Systematic Review and Meta-Analysis Protocols (PRISMA-P)^13^ and registered in the International Prospective Register of Systematic Reviews (PROSPERO) database under number CRD42025638069. This systematic review was reported following the Preferred Reporting Items for Systematic Reviews and Meta-Analyses (PRISMA)^14^ and conducted according to the JBI Manual.^15^

### Research question and eligibility criteria

The review was designed to answer the following question: “What are the dimensional changes in the alveolar ridge in patients undergoing tooth extraction who received PRF for ridge preservation, compared to a control group, as assessed by CBCT?” following the PICO framework: P (population), I (intervention), C (comparison), and O (outcome).

### Inclusion criteria

**P** (Population): patients undergoing tooth extraction;

**I** (Intervention): use of PRF for alveolar ridge preservation post-extraction;

**C** (Comparison): use of blood clot for control group;

**O** (Outcome): dimensional changes in alveolar ridge measured by CBCT;

Study design: randomized clinical trials;

There were no restrictions on publication language or year.

### Exclusion criteria

• Studies not related to the topic;
• Studies that used bone grafts in association with PRF to preserve alveolar ridge post-extraction;
• Studies that used only clinical methods (e.g., periodontal probe), radiographs, digital scanning, or histological analysis to measure dimensional changes in alveolar ridge;
• Studies with patients undergoing third molars extraction;
• Studies with overlapping samples (e.g., same authors and samples, but different years and journals of publication), in these cases being considered the most recent study that best describes methodology and results;
• Books, book chapters, case reports, case series, event annals, editorials, letters to the editor, literature reviews, qualitative studies and animal studies.

### Sources of information, search, and selection of studies

The electronic searches were performed on January 2024 in Embase, Latin American and Caribbean Health Science Literature (LILACS), Brazilian Bibliography of Odontology (BBO), MedLine (via PubMed), and SciELO; and the Scopus and Web of Science citation databases. The Google Scholar and OpenGrey databases partially captured the “gray literature”. The references list of eligible studies was also manually revised to search for other studies in potential. These steps were performed to minimize the selection bias. The MedLine search was constantly updated with electronic alerts until January 2025. The search descriptors were selected according to the MeSH (Medical Subject Headings), DeCS (Health Sciences Descriptors), and Emtree (Embase Subject Headings) resources. The Boolean operators “AND” and “OR” promoted several combinations among the descriptors, respecting the syntax rules of each database. Table 1 shows more details of search strategies and databases.

**Table 1:** Database search strategies.

Databases	Search strategies (January 2024) and Update (January 2025)
Main databases	
Embase https://www.embase.com	('alveolar bone atrophy' OR 'alveolar bone loss'/exp OR 'alveolar bone loss' OR 'bone remodeling'/exp OR 'bone remodeling' OR 'alveolar ridge'/exp OR 'alveolar ridge' OR 'bone healing'/exp OR 'bone healing' OR 'bone resorption'/exp OR 'bone resorption' OR 'dimensional changes') AND ('alveolar ridge preservation'/exp OR 'alveolar ridge preservation' OR 'tooth extraction'/exp OR 'tooth extraction' OR 'tooth loss'/exp OR 'tooth loss') AND ('platelet-rich fibrin'/exp OR 'platelet-rich fibrin' OR 'l-prf'/exp OR 'l-prf' OR 'growth factors'/exp OR 'growth factors' OR 'leukocyte-platelet-rich fibrin' OR 'platelet concentrates')
LILACS and BBO http://lilacs.bvsalud.org/	(("alveolar bone atrophy" OR "alveolar bone loss" OR "bone remodeling") AND ("alveolar ridge preservation" OR "tooth extraction") AND ("Platelet-rich fibrin" OR "L-PRF" OR "platelet concentrates")) AND (db:("LILACS"))
MEDLINE (via PubMed) http://www.ncbi.nlm.nih.gov/pubmed	("alveolar bone loss"[Mesh] OR "bone remodeling"[Mesh] OR "bone healing"[tw] OR "bone resorption"[tw] OR "dimensional changes"[tw]) AND ("alveolar ridge preservation"[tiab:∼0] OR "tooth extraction"[Mesh] OR "tooth loss"[tw]) AND ("platelet-rich fibrin"[Mesh] OR "L-PRF"[tw] OR "growth factors"[tw] OR "leukocyte-platelet-rich fibrin"[tw] OR "platelet concentrates"[tw])
sSciELO https://scielo.org/	(("alveolar bone atrophy" OR " alveolar bone loss" OR "bone remodeling") AND ("alveolar ridge preservation" OR "tooth extraction") AND ("Platelet-rich fibrin" OR "L-PRF" OR "platelet concentrates"))
Scopus http://www.scopus.com/	("alveolar bone atrophy" OR "alveolar bone loss" OR "bone remodeling" OR "alveolar ridge" OR "Bone healing" OR "bone resorption" OR "dimensional changes") AND ("alveolar ridge preservation" OR "tooth extraction" OR "tooth loss") AND ("Platelet-rich fibrin" OR "L-PRF" OR "growth factors" OR "leukocyte-platelet-rich fibrin" OR "platelet concentrates")
Web of Science http://apps.webofknowledge.com/	("alveolar bone atrophy" OR "alveolar bone loss" OR "bone remodeling" OR "alveolar ridge" OR "Bone healing" OR “bone resorption" OR "dimensional changes") AND ("alveolar ridge preservation" OR "tooth extraction" OR "tooth loss") AND ("Platelet-rich fibrin" OR "L-PRF" OR "alveolar" OR "growth factors" OR "leukocyte-platelet-rich fibrin" OR "platelet concentrates")
Gray literature	
Google Scholar https://scholar.google.com/	(("alveolar bone atrophy" OR "alveolar bone loss" OR "bone remodeling") AND ("alveolar ridge preservation" OR "tooth extraction") AND ("Platelet-rich fibrin" OR "L-PRF" OR "platelet concentrates))
OpenGrey http://www.opengrey.eu/	(("alveolar bone loss" OR "bone remodeling" OR “alveolar ridge preservation" OR " tooth extraction") AND ("Platelet-rich fibrin" OR "L-PRF" OR "platelet concentrates))

The obtained results were exported to the EndNote Web™ software (Clarivate™ Analytics, Philadelphia, USA), in which duplicates were removed automatically, and the remaining ones were removed manually. The other results were exported to Rayyan QCRI (Qatar Computing Research Institute, Doha, Qatar),^16^ for the study selection phase. The manual analysis of the gray literature occurred simultaneously and fully using Microsoft Word™ 2010 (Microsoft™ Ltd., Washington, USA).

Before selecting the studies, two reviewers performed a calibration exercise in which they discussed the eligibility criteria and applied them to a sample of 20% of the retrieved studies to determine inter-examiner agreement. The selection started after reaching an adequate level of agreement (Kappa ≥0.81), and occurred in three phases.

In the first phase, two eligibility reviewers (ACM and DMS) methodically analyzed the titles of the studies independently. A third examiner (CMM) investigated and solved disagreements between the reviewers. Titles unrelated to the topic were eliminated in this phase as well as abstracts in the second phase, respecting the eligibility criteria. In the third phase, the full texts of the preliminarily eligible studies were obtained and evaluated. If the full texts were not found, a bibliographic request was made to the library database (COMUT), and an e-mail was sent to the corresponding authors to obtain the texts.

### Data collection

A calibration exercise was performed before data extraction to ensure consistency between the reviewers, in which the data from three eligible study was extracted jointly. After the calibration, two reviewers (ACM and DMS) extracted the data from the eligible studies, independently and blinded. A third reviewer (CMM) analyzed the conflicts in cases of disagreement about data extraction.

The following data were extracted from the articles: study characteristics (author, year, title, journal of publication, impact factor using Journal Citation Reports 2025, study country, study design, type of randomization, ethical criteria, funding, and conflict of interest), sample characteristics (sample size, sex, age, eligibility criteria, and setting), data collection and processing (surgical sites, surgical technique, PRF type, PRF preparation protocol, wound closure technique, outcome measurement method), and main results (objectives, vertical and horizontal changes in alveolar ridge, percentage of repaired alveoli, final bone density, and main outcomes). In case of incomplete or insufficient data, the corresponding authors were contacted via e-mail up to three times at weekly intervals.

### Risk-of-bias assessment

Two reviewers (ACM and DMS) independently assessed the risk of bias in the selected studies using the Cochrane Collaboration Risk of Bias tool (version 2.0) (RoB2) for RCTs.^17^ This instrument consists of five domains: bias from the randomization process, bias due to deviations from intended interventions, bias from missing outcome data, bias in outcome measurement, and bias in the selection of reported results.

The evaluation of each domain followed the algorithms proposed by the RoB2 manual.^17^ Any disagreements between the reviewers were resolved by discussing and consulting with a third reviewer (CMM).

### Data synthesis and meta-analysis

The data collected from the selected studies were organized in spreadsheets on Microsoft Excel™ 2019 (Microsoft™ Ltd., Washington, USA) and described narratively (qualitative synthesis). The quantitative results of vertical and horizontal changes in alveolar ridge, percentage of repaired alveoli, and final bone density were described.

The meta-analysis was conducted to compare the effect of PRF with control/placebo on alveolar ridge preservation after tooth extraction. Continuous outcomes were analyzed considering dimensional bone changes assessed at different measurement levels, including 1, 3, and 5 mm, as well as bone loss in width and height, separately for the buccal and palatal aspects, when available.

For each comparison, means, standard deviations, and sample sizes were extracted from the PRF and control/placebo groups. The effect size was calculated as the standardized mean difference corrected for small-sample bias, expressed as Hedges’ g, using exact formulae for bias correction. Before data pooling, outcomes were oriented in the same direction to ensure consistent interpretation of the treatment effect across studies.

Pooled estimates were obtained using the inverse-variance method. Given the expected clinical and methodological heterogeneity among studies, a random-effects model was applied. Between-study variance was estimated using the Restricted Maximum-Likelihood (REML) estimator. Confidence intervals for τ² and τ were calculated using the Q-profile method. Statistical heterogeneity was assessed using Cochran’s *Q*-test, τ², and *I*², with *I*² calculated based on the Q statistic. To provide more conservative inference in the presence of a limited number of studies, the Hartung-Knapp adjustment was applied to the confidence intervals and significance tests of the random-effects model.

Mixed-effects meta-regression analyses were conducted exploratorily to investigate potential sources of between-study heterogeneity. The assessment time point, risk of bias of the included studies, and type of PRF were evaluated as potential moderators. Meta-regression models were fitted using the REML estimator for τ², while retaining the Hartung-Knapp adjustment for coefficient testing. For categorical moderators, indicator variables were created, with one category selected as the reference group. The proportion of heterogeneity explained by the moderators was expressed as the meta-regression R², whereas residual heterogeneity was evaluated using τ², residual I², and the Q test for residual heterogeneity.

All analyses were performed in the R environment using dedicated packages for meta-analysis and meta-regression. A *p*-value lower than 0.05 was considered statistically significant.

### Certainty of evidence

Two reviewers (WAV and DMS) assessed the certainty of the evidence using the Grading of Recommendations, Assessment, Development, and Evaluation (GRADE) approach. This assessment was based on study design, risk of bias, inconsistency, indirectness, imprecision, and other relevant considerations. The certainty of the evidence was categorized as high, moderate, low, or very low.

## Results

### Study selection

During the first phase of study selection, 2843 results were retrieved from eight electronic databases, including grey literature sources. After the removal of duplicates and screening of titles and abstracts, only 49 studies were selected for full-text assessment, of which 36 were excluded (Supplementary Material 1). The references of the potentially eligible studies were carefully reviewed, but no additional articles were identified. Finally, 13 studies^7^^,^^18^, ^19^, ^20^, ^21^, ^22^, ^23^, ^24^, ^25^, ^26^, ^27^, ^28^, ^29^ were included in the systematic review (Fig. 1).

**Fig. 1:**
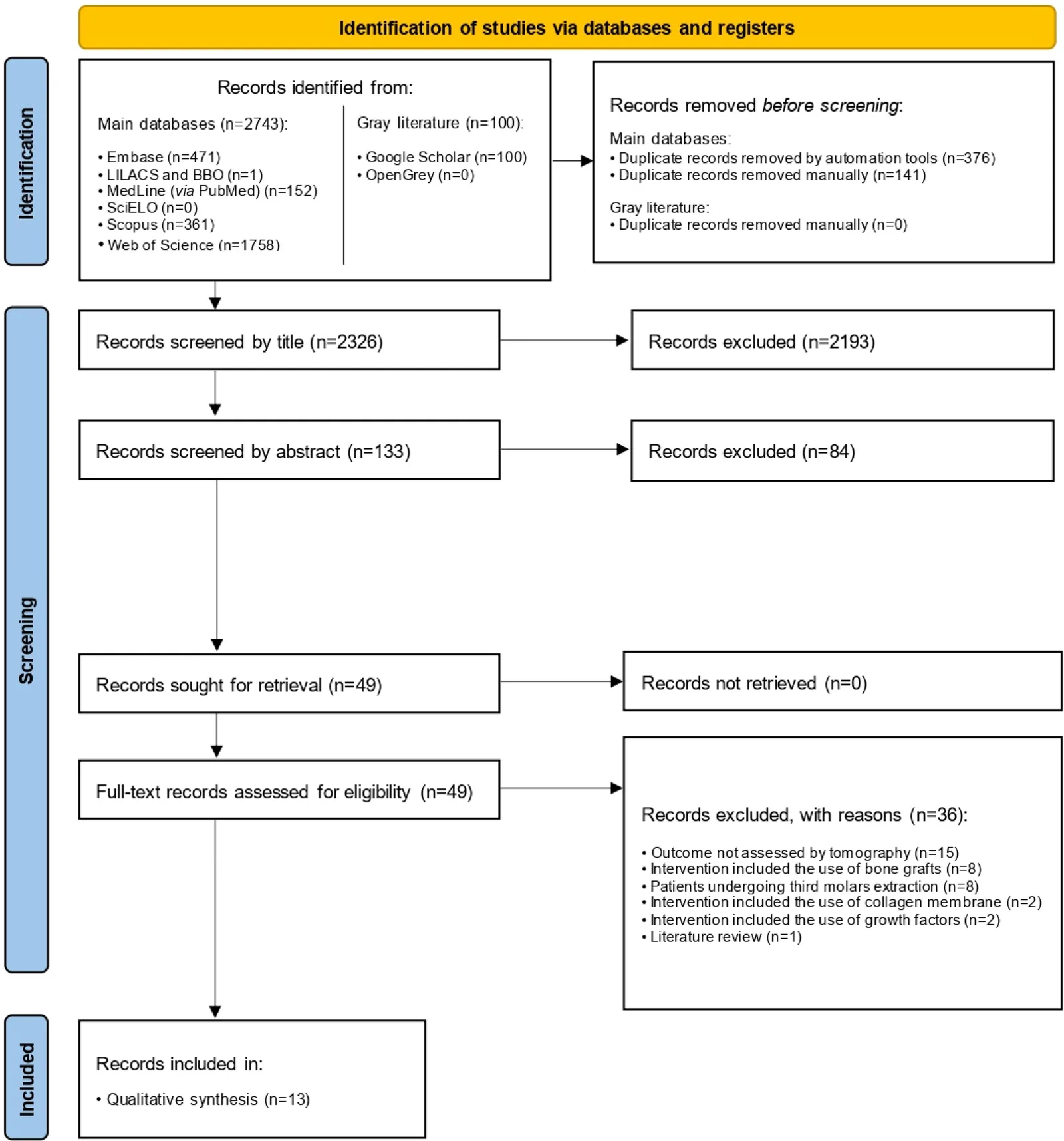
Flowchart of study selection.

### Main characteristics of eligible studies

The studies were published between 2016 and 2024. Of these, eight were conducted in Asia,^19^^,^^20^^,^^22^^,^^24^^,^^26^, ^27^, ^28^, ^29^ two in South America,^7^^,^^18^ two in Europe,^23^^,^^25^ and one in Africa.^21^ The total sample included 362 analyzed participants and 599 extraction sockets. Participants’ age across the studies ranged from 18- to 64-years. Additional results are presented in Table 2.

**Table 2:** Main characteristics of eligible studies.

Author, year (Country)	Study design	Sample size		PRF Protocol		
**Journal of publication (JCR impact factor)**	**^$^ Funding** ^#^ Ethical criteria * Conflict of interest	**Participants (n) | Alveoli (n)** **Sex distribution (♂, ♀)** **Age range / Mean age**	**Surgery description (sites, technique)**	**PRF type**	**PRF preparation**	**Wound closure technique**
Temmerman et al., 2016 (Chile) Journal of Clinical Periodontology (6.8)	RCT, split-mouth ^$^ None ^#^ Ethical Committee of the KU Leuven, UZ Leuven University Hospitals * None	n = 22 | n = 44 (♂ = 15, ♀ = 7) > 18yo / 54±11yo	Symmetrical tooth extraction sites for control and testing. Flapless and atraumatic surgery.	PRF clots L-PRF membranes	2700 rpm for 12 min	2-to-5 PRF clots covered by 2-to-3 L-PRF membranes and stabilized with a crossed horizontal mattress suture followed by individual sutures without attempt to close the wound.
Srinivas et al., 2018 (India) Annals of Maxillofacial Surgery (NF)	RCT, split-mouth ^$^ None ^#^ Institutional Ethics Committee NS * None	n = 30 | n = 60 Sex distribution NR 20‒50yo / Mean age NR	Placement of PRF on one side and the other side should be considered the control group. Flapless and atraumatic surgery.	PRF	3.000 rpm for 10 min	The alveoli of the control group were debrided and sutured, while the alveoli of the treated group were debrided and the PRF was grafted and received simple interrupted sutures.
Zhang et al., 2018 (China) Experimental and Therapeutic Medicine (2.3)	RCT, parallel groups ^$^ Science and Technology Development Exhibition Innovation Fund of Pudong New District ^#^ Ethics Committee of East Hospital Affiliated to Tongji University * NR	n = 28 | n = 56 (♂ = 14, ♀ = 14) 20‒40yo / Mean age NR	The experimental group was implanted with PRF membrane, whereas the control group was not. Minimally invasive tooth extraction.	PRF	400g for 10 min	The prepared PRF membranes were cut into a suitable size and implanted into the extraction socket, and finally sutured.
Taha, 2019 (Egypt) Egyptian Journal of Oral and Maxillofacial Surgery (NF)	RCT, parallel groups ^$^ None ^#^ NR * None	n = 27 | n = 42 (♂ = 8, ♀ = 19) 23‒49yo / 37.5±5yo	The experimental group was implanted with PRF membrane, whereas the control group was not. Minimally invasive tooth extraction.	PRF	3.000 rpm for 10 min	PRF was placed solely after extraction, and it was stabilized using 4‒0 polyglactin (Vicryl) suture.
Badakhshan et al., 2020 (Iran) Journal of Long-Term Effects of Medical Implants (NF)	RCT, split-mouth ^$^ NR ^#^ Ethics committee at the Shahid Beheshti Dental School * NR	n = 19 | n = 44 (♂ = 9, ♀ = 10) 25‒65yo / Mean age NR	The tooth was extracted; the dental socket was curetted and washed. L-PRF was placed in the socket containing fresh blood. Flapless and atraumatic surgery.	L-PRF	2700 rpm for 12 min	Closed with external horizontal crossed sutures.
Canellas et al., 2020 (Brazil) Journal of Cranio-Maxillo-Facial Surgery (2.1)	RCT, parallel groups ^$^ None ^#^ Ethics Committee of Pedro Ernesto Hospital, Rio de Janeiro State University, Brazil * None	n = 48 | n = 48 (♂ = 21, ♀ = 27) 18‒69yo / 44.8yo	Extraction, irrigation with saline solution, and placement of L-PRF in the treated group. Flapless and atraumatic surgery.	L-PRF	2.700 rpm for 12 min	1 membrane and 4 L-PRF sockets were used. All patients received sutures without primary closure.
Castro et al., 2021 (Belgium) Journal of Clinical Periodontology (6.8)	RCT, split mouth ^$^ NR ^#^ Ethical Committee of the KU Leuven, UZ Leuven University Hospitals * None	n = 20 | n = 60 (♂ = 5, ♀ = 15) Age range NR / Mean age NR	After extraction, the sockets were curetted and filled with L-PRF, A-PRF in the treated groups. Flapless and atraumatic surgery.	L-PRF A-PRF	2700 rpm (RCFclot: 408g) for 12 min	2 or 3 membranes were placed in the alveoli treated with L-PRF or A-PRF and compressed with an amalgam plunger. An envelope was created on the buccal and palatal sides to create space for 1–2 folded membranes placed in this envelope to seal the alveolus. A horizontal crisscross mattress suture was applied.
Wang et al., 2022 (China) Journal of Clinical Periodontology (6.8)	RCT, split mouth ^$^ Faculty of Dentistry; University of Hong Kong; and European Research Group on Periodontology, Switzerland ^#^ Institutional Review Board of the University of Hong Kong/Hospital * Two authors reported financial support from various dental material brands outside the scope of the study	n = 18 | n = 36 (♂ = 9, ♀ = 9) 18–70yo / 41.67±12.73yo	The tooth was gently luxated, the socket was curetted and irrigated with saline. Flapless and atraumatic surgery.	L-PRF	408g for 12 min	The L-PRF treated group was filled with plugs/membranes. A single cross suture was placed to stabilize the L-PRF membrane in the socket.
Abad et al., 2023 (Spain) Clinical Implant Dentistry and Related Research (4.0)	RCT, parallel groups ^$^ None ^#^ Local ethics committee NS * None	n = 27 | n = 27 Sex distribution NR 18‒60yo / Mean age NR	After extraction, the treated group received L-PRF. Minimally invasive tooth extraction.	L-PRF	2700 rpm (RCFclot: 408g) for 12 min	4 plugs and 4 membranes of L-PRF. Full-thickness flaps were raised in the lingual and buccal regions. The membranes were fixed with a horizontal cross suture.
Assadi et al., 2023 (Iran) **Journal of Osseointegration (NF)**	RCT, split mouth ^$^ Dentistry Faculty of Tabriz Medical Science University, Iran ^#^ Research Ethics Committees of Tabriz University of Medical Sciences * None	n = 39 | n = 78 (♂ = 18, ♀ = 21) 19‒64yo / Mean age NR	After tooth removal, the extraction sockets were completely debrided and after curettage, the sockets were rinsed with saline solution. Minimally invasive tooth extraction.	CGF	2.700 rpm for 2 min, 2.400 rpm for 4 min, 2.700 rpm for 4 min, 3.000 rpm for 3 min and desaccelaration until stop for 36 sec	The CGF Group membrane was cut into a smaller shape so that it could fit into the extraction socket and was then stabilized with tight non-resorbable sutures.
Kassar & Heshmeh, 2023 (Syria) Journal of Stomatology (NF)	RCT, split mouth ^$^ nr ^#^ Scientific committee of the Faculty of Dentistry, Damascus University * None	n = 20 | n = 40 Sex distribution NR 18‒60yo / Mean age NR	The alveoli were cleaned after extraction by filling an extraction socket with L-PRF and the other alveolus remaining empty. Minimally invasive tooth extraction.	L-PRF	2.700 rpm for 12 min	The L-PRF membrane was stabilized with crossed sutures.
Alasqah et al., 2024 (Saudi Arabia) Medicina (2.4)	RCT, split mouth ^$^ Yes ^#^ Institutional Review Board of Riyadh Elm University * None	n = 40 | n = 40 Sex distribution NR 18‒60yo / Mean age NR	After tooth removal, the socket was curetted and cleaned with saline. Minimally invasive tooth extraction.	L-PRF	2500 rpm for 10 min	L-PRF plugs and membranes were placed in the socket and sutured in a figure-of-eight fashion.
Mousavi et al., 2024 (Iran) Oral and Maxillofacial Surgery (1.8)	RCT, split mouth ^$^ Yes. ^#^ Ethics committee of Tehran University of Medical Sciences * None	n = 24 | n = 24 (♂ = 3, ♀ = 21) Age range NR L-PRF: 44.33±14.2yo CTL: 37.5±6.5yo	The extraction socket was curetted and rinsed with normal saline. Minimally invasive tooth extraction.	L-PRF	2.700 rpm (RCFclot 408g) for 12 min	The control group received L-PRF plugs, while the control group received only a figure-of-eight suture over the socket.

NF, Not found; NS, Not Specified in the Study; NR, Not Reported in the study; RCT, Randomized Clinical Trial; PRF, Platelet Rich Fibrin; L-PRF, Leucocyte-Platelet Rich Fibrin; A-PRF, Advanced Platelet Rich Fibrin; CGF, Concentrated Growth Factor; rpm, Rotation per minute; min: Minutes; sec, Seconds.

### Risk of individual bias in the studies

Among the 13 studies, six^18^, ^19^, ^20^, ^21^, ^22^^,^^26^ were classified as a “high risk of bias”, and the remaining seven studies^7^^,^^23^, ^24^, ^25^^,^^27^, ^28^, ^29^ had “some concerns”. Four studies^19^, ^20^, ^21^, ^22^ presented bias in randomization and allocation sequence concealment. All studies presented bias related to blinding, one of them^7^ even presented controversial parts of the article on the blinding of surgeons. Three studies^26^, ^27^, ^28^ lacked information on their surgeons, such as calibration and technical reports. While seven studies^18^, ^19^, ^20^, ^21^^,^^26^, ^27^, ^28^ did not present details on their outcome assessors. Two studies^20^^,^^26^ had missing data on follow-up losses and methods to address them. Finally, one study^25^ reported significant baseline differences between study groups related to the presence of more alveoli with buccal bone loss in the intervention group. Fig. 2 shows the individual assessment of each study. Fig. 3 presents a summary plot of the risk of bias for each bias domain.

**Fig. 2:**
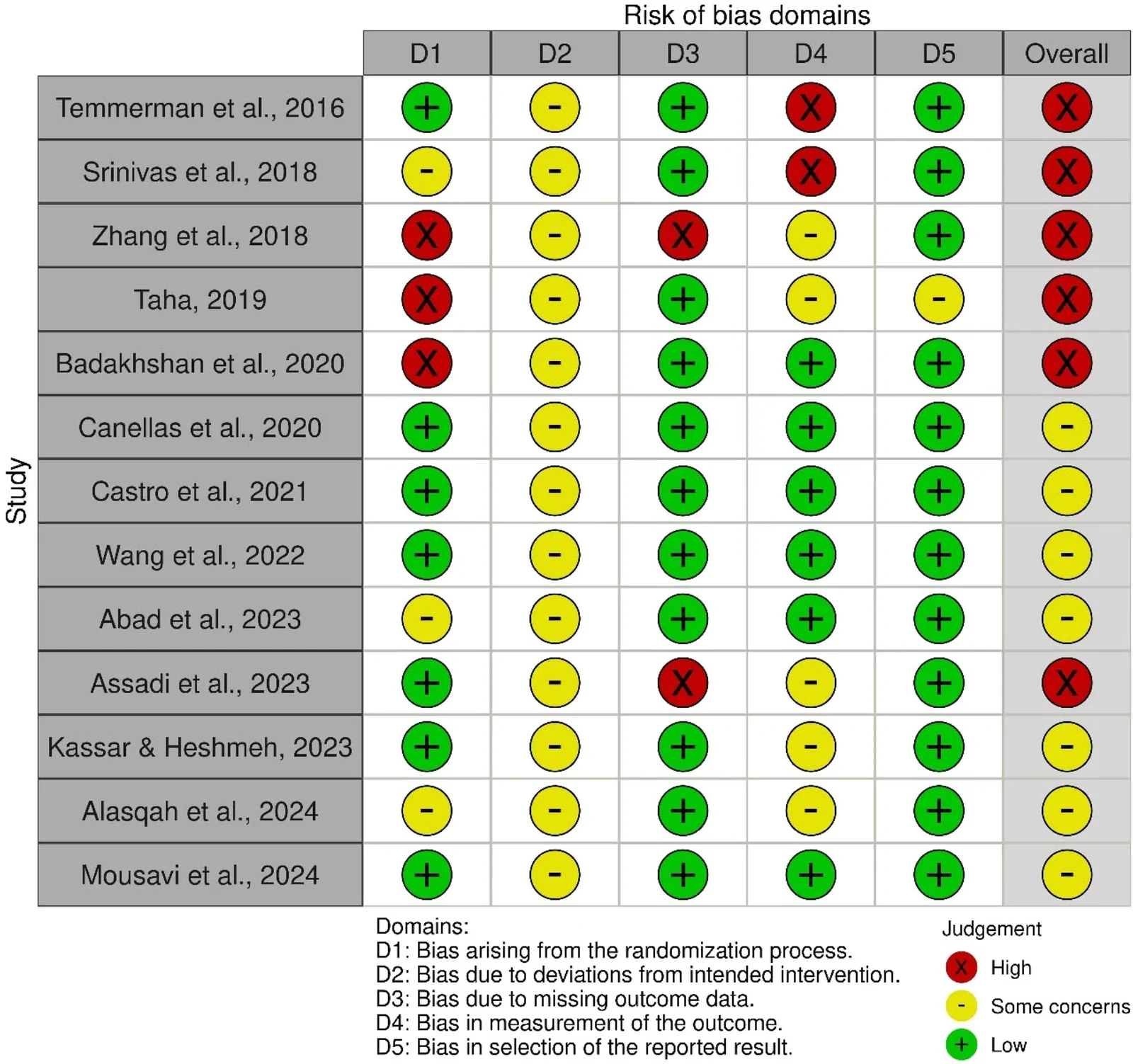
Risk of bias’ results for each eligible study.

**Fig. 3:**
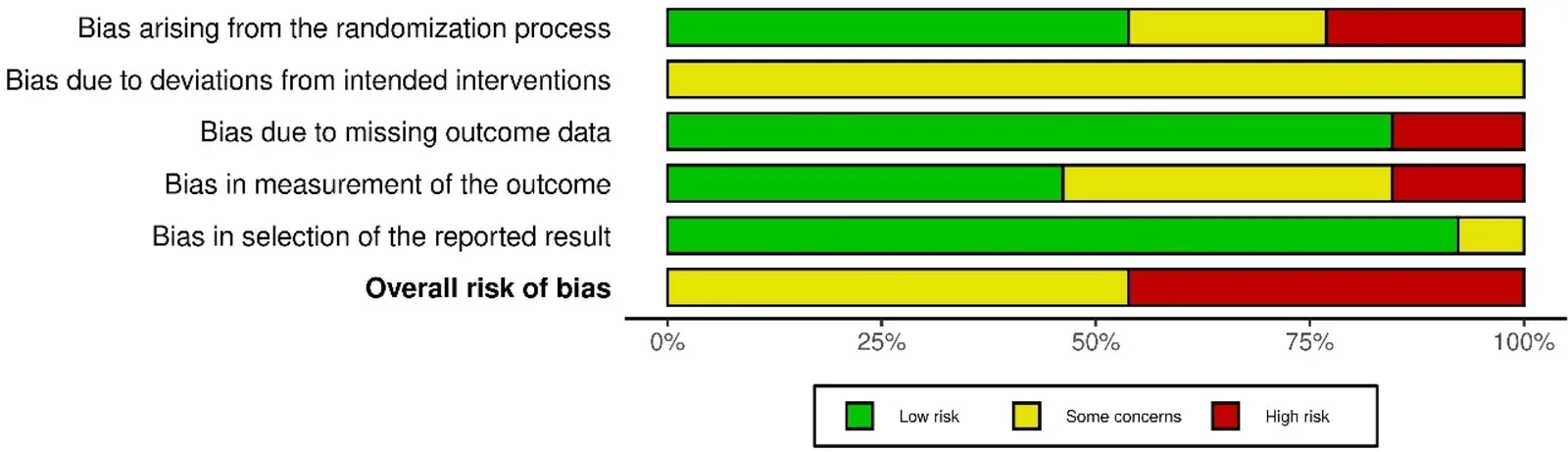
Summary plot of risk of bias’ results.

### Individual results of eligible studies

Of the 13 eligible studies, seven^7^^,^^18^^,^^19^^,^^21^^,^^22^^,^^26^^,^^27^ reported a positive association between the use of PRF ‒ applied through different protocols ‒ and alveolar ridge preservation, and five of them were in the high risk-of-bias group.^18^^,^^19^^,^^21^^,^^22^^,^^26^ In contrast, the remaining studies^20^^,^^23^, ^24^, ^25^^,^^28^^,^^29^ did not observe significant benefits of PRF in minimizing post-extraction bone resorption, with only one study of them presenting high risk-of-bias.^20^

Regarding alveolar ridge width (horizontal dimension), results varied widely across both control and PRF-treated groups. Some studies indicated that PRF contributed to the preservation of ridge width at follow-up periods ranging from 90- to 180-days. For example, among high risk-of-bias studies, Badakhshan et al. (2020)^22^ reported an average ridge width of 7.66 mm in the PRF group compared to 7.54 mm in the control group after 90-days, suggesting a potential positive effect of PRF. However, among studies with some bias concerns, Abad et al. (2023)^25^ observed a significant reduction in ridge width in the PRF group (-6.37 mm), highlighting the inconsistency of the findings and reinforcing the controversy surrounding PRF’s effectiveness in this dimension.

Alveolar ridge height (vertical dimension) also showed similar variability between groups. Heights in the control groups ranged from 12.28 mm to 19.0 mm, while in the PRF groups values ranged from 12.28 mm to 19.14 mm.^19^^,^^26^ These results, among high risk-of-bias studies, suggest that PRF may contribute to the maintenance of ridge height, although not always with statistical significance.

Final bone density, although less frequently reported, was addressed in a few studies, which indicated enhanced bone regeneration in the PRF-treated groups.^27^^,^^29^ Additionally, a short-term reduction in postoperative pain associated with PRF use was observed.^28^ However, data on bone density and postoperative pain remain inconsistent, due to significant methodological variability among studies, which may affect the comparability of the findings (Table 3, Table 4).

**Table 3:** Main outcomes of eligible studies.

Author, year	Main outcomes
Temmerman et al., 2016	The use of L-PRF as a socket filling material to achieve preservation of horizontal and vertical ridge dimension at three months after tooth extraction is beneficial. Furthermore, the use of L-PRF results in less postoperative discomfort and pain for the patients, especially during the early phases of healing.
Srinivas et al., 2018	An appreciable amount of bone regeneration was observed in the experimental group when compared to the control.
Zhang et al., 2018	PRF used in extraction sockets promoted local healing of the soft tissues of the gingiva and reduced the postoperative pain response; however, the effect of PRF on reducing alveolar bone resorption was not significant. However, PRF was able to increase the quality of new bone and increase the rate of bone formation due to the concentration of growth factors.
Taha, 2019	Within six months, the control showed a higher value of bone loss in the buccal table when compared to the PRF.
Badakhshan et al., 2020	Application of L-PRF in tooth extraction sockets significantly decreased reductions in bone height and buccolingual diameter compared to control sockets.
Canellas et al., 2020	L-PRF provides significant benefits in terms of alveolar preservation, decreasing horizontal resorption by 1 mm and 3 mm below the alveolar crest. In addition, it improves total bone volume after 3-months.
Castro et al., 2021	PRF matrices failed to reduce dimensional changes after multiple tooth extractions in the premaxilla. After 3-months of healing, both PRF matrices radiographically showed significant superiority for filling the socket. Histologically, they appeared to accelerate new bone formation.
Wang et al., 2022	L-PRF increased the concentrations of growth factors in the alveoli, without altering the pattern observed in unassisted healing.
Abad et al., 2023	Alveolar preservation with L-PRF did not minimize bone resorption that occurs after tooth extraction
Assadi et al., 2023	It can result in significantly better pain control, soft tissue healing, and reduced bone loss compared to natural healing.
Kassar & Heshmeh, 2023	The use of L-PRF for the purpose of reducing post-extraction bone resorption and preserving the alveolar ridge is considered beneficial, cost-effective and recommended in a planned implant treatment.
Alasqah et al., 2024	L-PRF was effective in preserving alveolar ridge volume and reducing postoperative pain in extracted sockets.
Mousavi et al., 2024	L-PRF did not reduce the rate of ridge resorption in the vertical or horizontal dimensions of the extraction sockets.

PRF, Platelet Rich Fibrin; L-PRF, Leukocyte-Platelet Rich Fibrin.

**Table 4:** Main quantitative results of eligible studies.

Author, year	Alveolar width – CTL (Horizontal in mm)	Alveolar width – PRF (Horizontal in mm)	*p*-value	Alveolar height – CTL (Vertical in mm)	Alveolar height – PRF (Vertical in mm)	*p*-value	Repaired alveoli (%)	*p*-value	Final bone density (socket site)	p-value
Temmerman et al., 2016	HRW 1-mm 90-days = -5.19±2.42 HRW 3-mm 90-days = -14.51±19.6% HRW 5-mm 90-days = -4.47±4.89 Total loss (in %) = -51.92±40.31%	HRW 1-mm 90-days = -2.28±4.28 HRW 3-mm 90-days = -5.42±6.16 HRW 5-mm 90-days = -2.91±4.54 Total loss (in %) = -22.84±24.28%	0.005 0.03 NR 0.0004	B 90-days = -1.5±1.3 L/P 90-days = 0.7±0.8	B 90-days = 0.5±2.3 L/P 90-days = 0.4±1.1	0.0002 >0.5	CTL = 63.3% L-PRF = 94.7%	0.004	NR	N/A
Srinivas et al., 2018	NR	NR	N/A	CTL 24h = 14.68±4.32 CTL 90-days = 12.78±3.82	PRF 24h = 13.93±3.56 PRF 90-days = 12.28±3.84	0.466 0.615	NR	N/A	CTL 24h = 194.82 PRF 24h = 319.79 CTL 90-days = 295.87 PRF 90-days = 564.76	<0.001 for all
Zhang et al., 2018	a90-days = 2.07±1.67	a90-days = 1.05±0.77	NR	aB 90-days = 2.8±1,81 aL/P 90-days = 2.05±1.29	aB 90-days = 1.6±1.46 aL/P 90-days = 1±0.7	NR	NR	N/A	NR	N/A
Taha, 2019	a90-days = 1.49±0.6 a180-days = 2.03±0.7	a90-days = 1.48±0.9 a180-days = 2.24±1.06	>0.5 for all	B 90-days = 0.79±0.6 L/P 90-days = 0.95±0.78 B 180-days = 1.92±0.9 L/P 180-days = 1.12±1.2	B 90-days = 2.41±0.8 L/P 90-days = 1.46±0.7 B 180-days = 1.71±0.5 L/P 180-days = 0.96±0.52	>0.5 for all	NR	N/A	NR	N/A
Badakhshan et al., 2020	HRW0 immediate = 7.54±1.05 HRW0 90-days = 5.46±1.37 HRW 1-mm immediate = 7.66±0.76 HRW 1-mm 90-days = 6.27±0.77 HRW 3-mm immediate = 7.99±1.59 HRW 3-mm 90-days = 6.22±1.56	HRW0 immediate = 7.03±2.09 HRW0 90-days = 6.1±2.1 HRW 1-mm immediate = 7.31±2.27 HRW 1-mm 90-days = 6.69±2.34 HRW 3-mm immediate = 7.71±1.68 HRW 3-mm 90-days = 7.4±1.79	<0.0001 for all	B 0-days = 19±1.75 L/P 0-days = 17.1±3.12 B 90-days = 15.21±1.6 L/P 90-days = 14.1±2.46	B 0-days = 19.14±3.75 L/P 0-days = 17.93±4.35 B 90-days = 17.16±3.87 L/P 90-days = 16.56±4.15	<0.0001 for all	NR	N/A	NR	N/A
Canellas et al., 2020	HRW1 90-days = -2.27±1.21 HRW 3-mm 90-days = -1.67±1.1 HRW 5-mm 90-days= -1.08±1.04	HRW1 90-days = -0.93±0.93 HRW 3-mm 90-days = -0.85±0.81 HRW 5-mm 90-days = -0.67±0.56	0.0001 0.005 0.094	B 90-days = -1.39±1.28 L/P 90-days = -1.24±1.15	B 90-days = -0.7±0.77 L/P 90-days = -0.67±0.9	0.028 0.064	NR	N/A	NR	N/A
Castro et al., 2021	HRW1 90-days = -2.2±1.1 HRW 3-mm 90-days = -1.7±0.8 HRW 5-mm 90-days = -1.4±0.8	L-PRF HRW 1-mm 90-days = -2.2±1 L-PPF HRW 3-mm 90-days = -1.8±1.7 L-PRF HRW 5-mm 90-days = -1.2±0.8 A-PRF HRW 1-mm 90-days = -2.2±0.9 A-PRF HRW 3-mm 90-days = -1.6±0.9 A-PRF HRW 5-mm 90-days = -1.2±0.8	>0.5 for all	B 90-days = -0.2±0.8 L/P 90-days = -1±0.9	L-PRF B 90-days = 0.2±1.2 L-PRF L/P 90-days = -1.1±0.9 A-PRF B 90-days = 0.2±1.1 A-PRF L/P 90-days = -1±0.8	>0.5 for all	CTL = 67.9%±19.2 L-PRF = 85.2%±22.9 A-PRF = 83.8%±18.4	L-PRF vs. A-PRF: 0.9 L-PRF vs. CTL: 0.005 A-PRF vs. CTL: 0.01	NR	N/A
Wang et al., 2022^b^	HRW 1-mm 150-days = 4.75±3.65 HRW 3-mm 150-days = 2.63±2.68 HRW 5-mm 150-days = 1.89±1.56	HRW 1-mm 150-days = 4.80±3.49 HRW 3-mm 150-days = 1.68±1.22 HRW 5-mm 150-days = 0.85±0.65	0.154 0.329 0.207	B 150-days = 0.44±0.84 L/P 150-days = 1.13±1.19	B 150-days = 0.89±1.22 L/P 150-days = 1±0.94	0.644 0.103	NR	N/A	NR	N/A
Abad et al., 2023	HRW 1-mm 120-days = -6.67±3.13 HRW 3-mm 120-days = -2.26±1.81 HRW 5-mm 120-days = -1.68±1.49	HRW 1-mm 120-days = -6.37±2.95 HRW 3-mm 120-days = -2.96±2.25 HRW 5-mm 120-days = -1.9±1.55	0.798 0.388 0.712	B 120-days = 2.73±2.19 L 120-days = 1.72±1.12	B 120-days = 1.91±1.43 L 120-days = 1.83±1.27	0.644 0.826	NR	N/A	NR	N/A
Assadi et al., 2023	Immediate = 5.82±0.91 60-days = 4.2±0.68	Immediate = 5.67±0.76 60-days = 5.47±0.71	0.432 <0.001	NR	NR	N/A	NR	N/A	NR	N/A
Kassar & Heshmeh, 2023	HRW0 120-days = 2.17±0.78 HRW 6-mm 120-days = 1.22±0.41	HRW0 120-days = 1.31±1.29 HRW 6-mm 120-days = 0.29±0.56	0.089 0.001	B 120-days = 1.65±1.09 L/P 120-days = 1.92±1.88	B 120-days = 0.46±0.29 L/P 120-days = 0.52±0.31	0.004 0.032	NR	N/A	NR	N/A
Alasqah et al., 2024	HRW 1-mm 0-days = 10.89±0.56 HRW 1-mm 90-days = 10.8 ±0.6	HRW 1-mm 0-days = 11.09±0.61 HRW 1-mm 90-days = 10.98±1.03	0.673	CTL 0-days = 10.52±1.95 CTL 90-days = 10.42±1.88	PRF 0-days = 10.02±1.04 PRF 90-days = 9.82±1.02	0.877	NR	N/A	NR	N/A
Mousavi et al., 2024	56-days = 0.58± 0.95	56-days = 1.3±0.66	0.04	B 56-days = 2.03± 1.36 L/P 56-days = 1.81± 1.28	B 56-days = 1.67±1.67 L/P 56-days = 1.62±1.78	0.57 0.76	NR	N/A	CTL = 576.25± 62.16 L-PRF = 620.42±59.94	0.09

NR, Not Reported; N/A, Not Applicable; HRW, Horizontal Ridge Width differences; B, Buccal plate; L/P, Lingual/Palatal plate; CTL, Control; PRF, Platelet-Rich Fibrin; L-PRF, Leukocyte-Platelet Rich Fibrin; A-PRF, Advanced Platelet-Rich Fibrin.

a Variation.

b Data provided by the author of the study.

### Meta-analysis: alveolar ridge width changes

Alveolar ridge width changes were analyzed at 1, 3, and 5 mm. Positive SMDs indicated results favoring PRF over control/placebo.

At 1 mm (Fig. 4A), the pooled effect did not show a statistically significant difference between PRF and control/placebo (SMD = 0.30; 95% CI: -0.21 to 0.81; *p* = 0.217), with substantial heterogeneity (I² = 74.2%; τ² = 0.374; Q = 34.86, *p* < 0.0001) and very low certainty of evidence. Subgroup analysis showed larger effects in studies at high risk of bias than in those with some concerns (SMD = 1.19 vs. 0.08; subgroup p = 0.009). This was confirmed by meta-regression, in which risk of bias significantly moderated the effect estimates (*p* = 0.035; R² = 48.39%). Type of PRF and follow-up time were not significant moderators for heterogeneity.

**Fig. 4:**
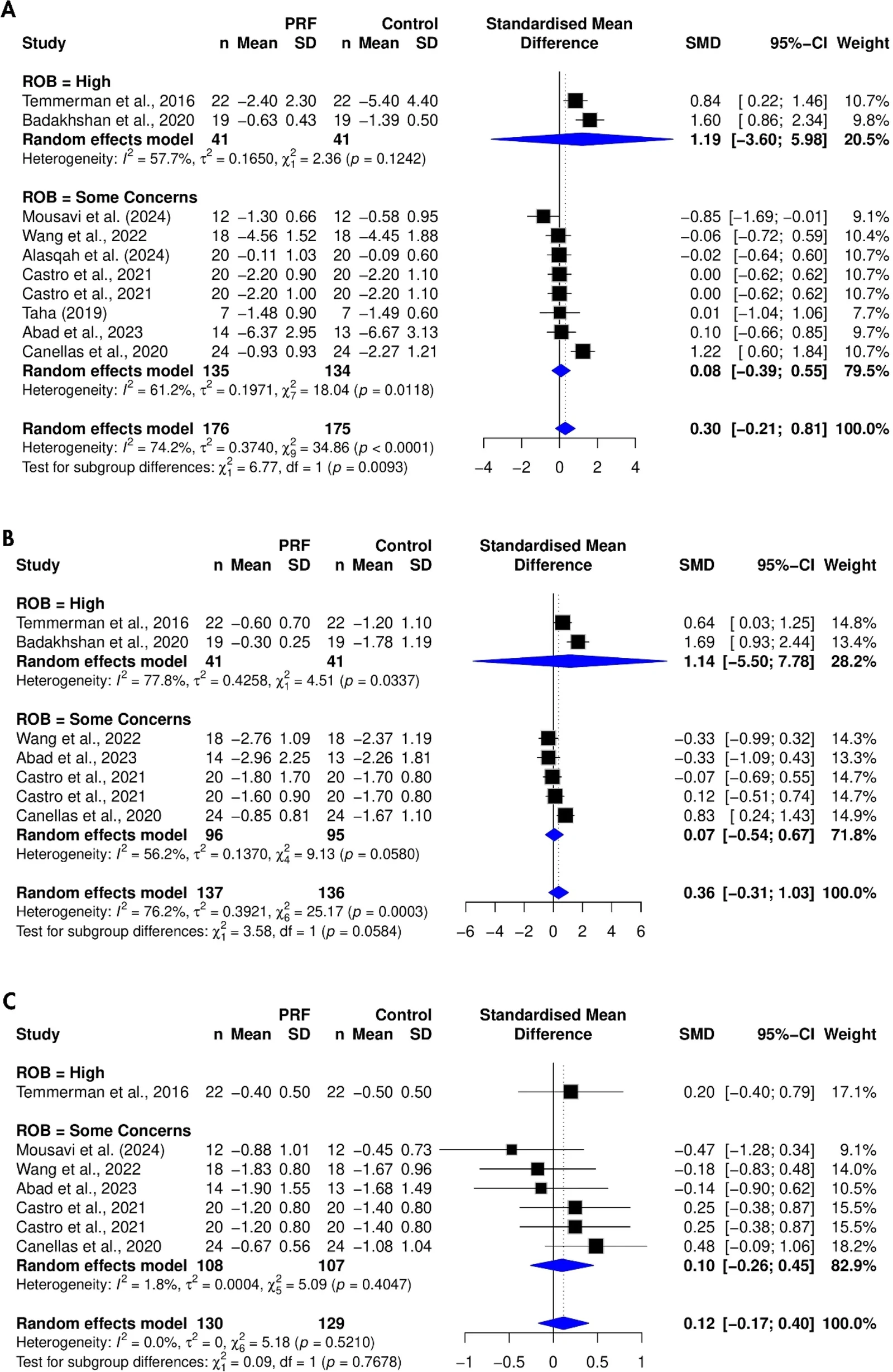
Forest plots comparing PRF and control for post-extraction alveolar bone width loss: (A) 1 mm, (B) 3 mm, and (C) 5 mm below the alveolar crest.

At 3 mm (Fig. 4B), no significant difference was observed between groups (SMD = 0.36; 95% CI: -0.31 to 1.03; *p* = 0.237), and heterogeneity was substantial (I² = 76.2%; τ² = 0.392; Q = 25.17, *p* = 0.0003). Studies at high risk of bias showed larger effects than those with some concerns, but the subgroup difference was not statistically significant (subgroup p = 0.058). Meta-regression suggested that risk of bias and follow-up time explained part of the heterogeneity (R² = 51.78% and 29.85%, respectively), although neither reached statistical significance. Type of PRF was not a significant moderator.

At 5 mm (Fig. 4C), the pooled effect was small and not statistically significant (SMD = 0.12; 95% CI: -0.17 to 0.40; *p* = 0.360), with no relevant heterogeneity (I² = 0.0%; τ² = 0.00; Q = 5.18, *p* = 0.521). Subgroup and meta-regression analyses did not identify significant effects of risk of bias, type of PRF, or follow-up time.

### Meta-analysis: buccal and palatal bone height changes

Buccal and palatal bone height changes were analyzed separately. Positive SMDs indicated results favoring PRF over control/placebo.

For buccal bone height (Fig. 5A), the pooled effect was not statistically significant (SMD = 0.43; 95% CI: -0.10 to 0.97; *p* = 0.103). Moderate-to-substantial heterogeneity was observed (*I*² = 67.6%; τ² = 0.322; Q = 30.85, *p* = 0.0006). Subgroup analysis according to risk of bias showed no significant difference between studies at high risk of bias and those with some concerns (subgroup *p* = 0.872). Similarly, meta-regression did not identify type of PRF, risk of bias, or follow-up time as significant moderators, and residual heterogeneity remained significant.

**Fig. 5:**
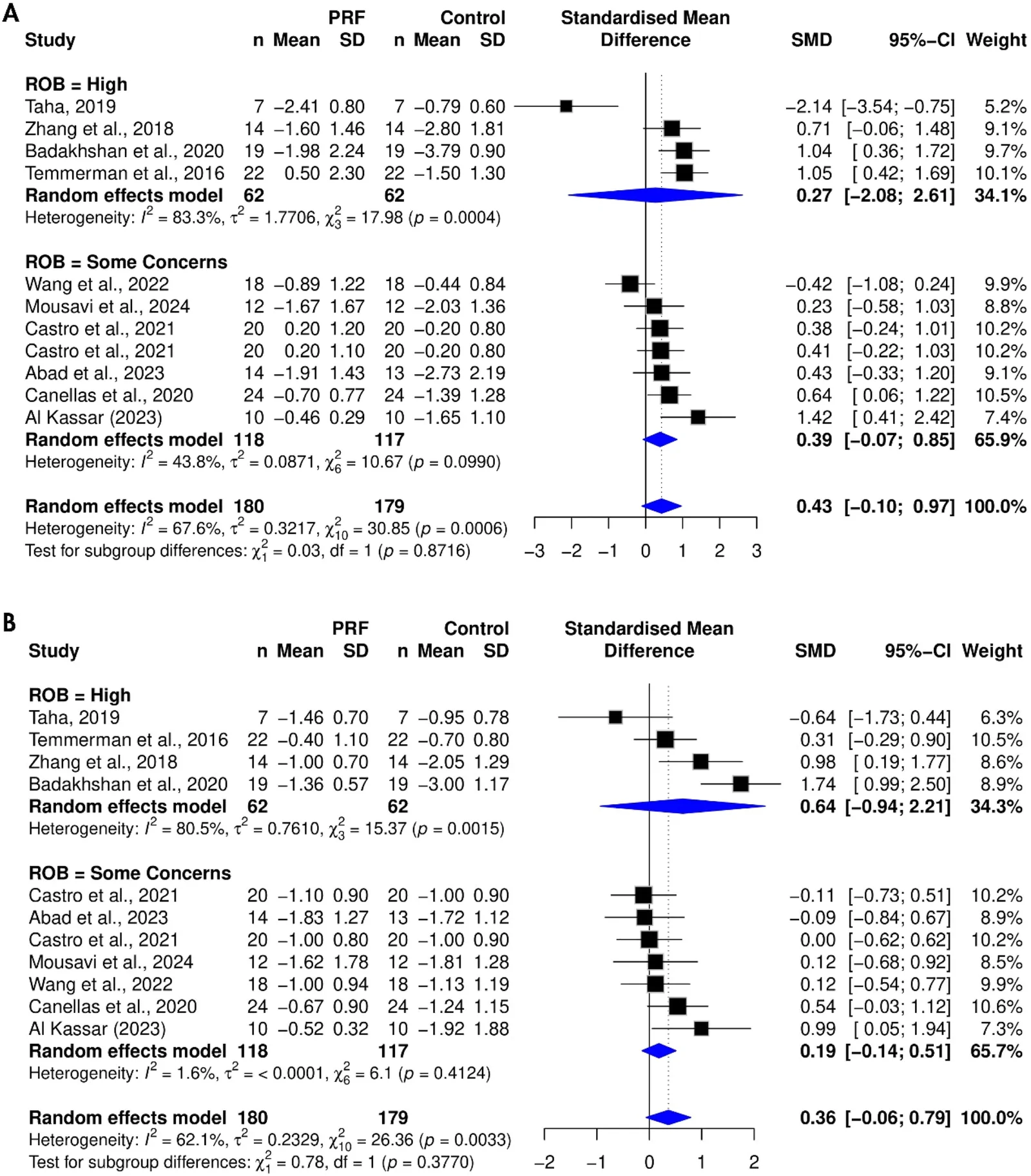
Forest plots comparing PRF and control for post-extraction alveolar bone height loss: (A) buccal plate loss and (B) palatal plate loss.

For palatal bone height (Fig. 5B), the difference was not statistically significant (SMD = 0.36; 95% CI: -0.06 to 0.79; *p* = 0.086). Heterogeneity was moderate-to-substantial (*I*² = 62.1%; τ² = 0.233; Q = 26.36, *p* = 0.003). Subgroup analysis by risk of bias showed no significant difference between subgroups (subgroup *p* = 0.377). In meta-regression, neither type of PRF nor follow-up time significantly influenced the effect estimates. Risk of bias explained 19.48% of the heterogeneity, but this association was not statistically significant (*p* = 0.252), and residual heterogeneity remained significant.

### Certainty of evidence

The certainty of evidence was analyzed according to the alveolar ridge width (horizontal) and height (vertical) changes. In all analyses, the certainty of evidence was downgraded because of samples lesser than 400 participants and confidence intervals crossing the threshold of null effect for appreciable benefit or appreciable harm (Table 5). Thus, both outcomes presented very low certainty of evidence.

**Table 5:** Summary of findings.

PRF versus Placebo			
	Relative effect (95% CI)	Number of participants (studies)	Certainty of evidence (GRADE)
*Outcome: Alveolar ridge width (horizontal changes)*			
1 mm	**SMD 0.08** (-0.39 to 0.55)	269 (7 RCT)	Very Low^a^
3 mm	**SMD 0.07** (-0.54 to 0.67)	191 (4 RCT)	Very Low^a^
5 mm	**SMD 0.12** (-0.26 to 0.45)	215 (5 RCT)	Very Low^a^
*Outcome: Alveolar ridge height (vertical changes)*			
Buccal	**SMD 0.43** (-0.10 to 0.97)	235 (6 RCT)	Very Low^a^
Palatal	**SMD 0.36** (-0.06 to 0.79)	235 (6 RCT)	Very Low^a^

RCT, Randomized Controlled Trials; C.I, Confidence interval; SMD, Standardized Mean Difference.

a Certainty of evidence was downgraded by one level because the total sample size was less than 400, and the 95% C.I. crosses the threshold of null effect for appreciable benefit or appreciable harm.

## Discussion

This systematic review assessed the available evidence in the literature regarding the effects of leukocyte- and platelet-rich fibrin on alveolar ridge preservation following tooth extraction, focusing exclusively on clinical studies that compared its effects with natural healing. To enable a more accurate evaluation of the isolated impact of PRF and its derivatives, all studies and study arms involving graft materials or membranes were excluded. Additionally, only studies employing CBCT for volumetric and dimensional measurements were included, ensuring greater precision in the assessment of alveolar changes.

Regarding alveolar bone preservation, two high risk-of-bias studies^18^^,^^22^ and two studies with some bias concerns^7^^,^^28^ reported reduced vertical and horizontal resorption of the alveolar ridge associated with the use of L-PRF. These findings are supported by a meta-analysis by Wang et al. (2024),^30^ which demonstrated that L-PRF had positive effects on bone formation and horizontal ridge width preservation. Additionally, Temmerman et al. (2016)^18^ reported a reduction in pain during the early stages of healing, possibly attributed to the supportive properties of L-PRF.^31^ In contrast, one high risk-of-bias study^26^ and four studies with some bias concerns^25^^,^^27^, ^28^, ^29^ did not observe significant effects on bone preservation, which may be explained by methodological variability among the included trials.

The individual results of eligible studies included in this review suggest partially favorable outcomes regarding the use of L-PRF for post-extraction alveolar preservation, particularly in reducing postoperative pain and enhancing soft tissue healing ‒ effects largely attributed to the sustained release of growth factors such as PDGF, TGF-β1, and VEGF from the fibrin matrix of L-PRF.^18^^,^^20^^,^^26^ In addition to growth factors, proteins such as fibrin, fibronectin, and vitronectin ‒ known for their role in osteoconduction via cell adhesion ‒ are also present. These components function as biological mediators that regulate cell proliferation, chemotaxis, and differentiation.^32^

Among the high risk-of-bias studies evaluating PRF, Zhang et al. (2018)^20^ reported improved gingival healing and bone quality, while others observed significant bone regeneration in PRF-treated groups.^19^^,^^21^ Castro et al. (2021)^23^ presented some bias concerns and found that although PRF did not prevent dimensional loss, it contributed to bone fill and new bone formation. A review by Al-Badran et al. (2023)^33^ concluded that PRF significantly reduced pain and accelerated tissue healing. These findings are further supported by studies showing that PRF promotes faster healing and reduces postoperative discomfort.^34^ The observed inconsistencies may be attributed to several factors, including variations in PRF centrifugation protocols, anatomical differences in extraction sites, differing evaluation periods (e.g., 30, 90, or 180-days), and small sample sizes in some studies. A study by Fujioka-Kobayashi et al. (2017)^35^ demonstrated that lower centrifugation speed and shorter duration may enhance the release of growth factors, thereby promoting tissue regeneration.

In the study by Assadi et al. (2023)^26^ ‒ which presented high risk of bias and was the only one to evaluate Concentrated Growth Factors (CGF) ‒ greater pain control and improved healing were reported, along with lower resorption rates and potential advantages over PRF. Experimental studies have shown that CGF forms a denser fibrin matrix, providing enhanced protection against bone resorption compared to PRF.^36^^,^^37^

In contrast, Azangookhiavi et al. (2024)^38^ found no statistically significant differences in mean vertical marginal bone loss or bleeding on probing between PRF and freeze-dried bone allograft groups. Similarly, a systematic review by Pan et al. (2019)^39^ reported that the effectiveness of PRF in horizontal ridge preservation was inconsistent; while some studies demonstrated a reduction in bone resorption with PRF, others did not.

Although individual clinical trials may suggest changes in alveolar ridge width (horizontal) and height (vertical) dimensions, caution must be exercised when interpreting these aggregated data. First, none of the eligible studies presented a low risk of bias, introducing methodological gaps that could distort the true treatment effect. In fact, subgroup analysis and meta-regression confirmed that studies at a high risk of bias presented a significant overestimation of effects. Second, the pooled estimates in the meta-analyses revealed a consistent lack of statistical significance for all alveolar ridge dimensional changes. Third, the certainty of evidence was rated as very low for all outcomes, driven by small sample sizes and wide confidence intervals crossing the null effect line, which ultimately restricts definitive inferences regarding either appreciable benefits or harms.

To add detail to this need for caution in data interpretation, some limitations should be acknowledged. The bias concerns among the included studies varied, with several presenting a high risk of bias or concerns related to randomization and blinding. It is important to note that this review included only studies comparing experimental and control groups, excluding experimental arms involving biomaterials such as bone grafts or membranes. Additionally, only tomographic evaluations were considered, due to their superior accuracy in assessing alveolar ridge dimensions ‒ setting this review apart from others,^32^ that included studies based on panoramic or periapical radiographs or subjective clinical assessments of healing. The specific focus on CBCT, while a strength, should also acknowledge its limitations. Accuracy is highly dependent on voxel size, beam hardening artifacts from adjacent dentition/implants, and the inherent inaccuracies of manual segmentation of thin, often dehisced, buccal plates.

A key strength of this review lies in its robust methodology and comprehensive reporting, characterized by strict eligibility criteria and a transparent analysis of individual results, risk of bias, pooled estimates, and certainty of evidence. By excluding interventions involving biomaterials and restricting the analysis to studies that employed tomographic measurements, the findings presented here more accurately reflect the isolated effects of PRF and L-PRF on alveolar healing. This approach contrasts with other systematic reviews^40^ that included less precise imaging techniques and combined therapeutic modalities. Furthermore, the granular details provided herein can guide future research, particularly in designing and standardizing more rigorous randomized clinical trials.

From a translational perspective, PRF and L-PRF may serve as adjunctive therapies in alveolar ridge preservation procedures, contributing to the acceleration of early soft tissue healing. However, their isolated effectiveness in preventing bone resorption remains controversial ‒ particularly when compared to bone substitutes, especially in cases involving substantial bone defects. While the standalone use of PRF and L-PRF appears beneficial for promoting healing and controlling postoperative pain, they present limitations in maintaining bone dimensions. Recent studies suggest that their efficacy is enhanced when combined with biomaterials. Given their autologous origin, low cost, and ease of preparation, PRF and L-PRF represent viable adjunctive options in clinical practice.

## Conclusions

Based on very low evidence, the application of PRF concentrates did not demonstrate appreciable benefits or appreciable harms for alveolar ridge preservation. However, these findings should be interpreted with caution due to data heterogeneity and bias concerns in primary studies. Thus, the evidence is insufficient to recommend its sole use as a predictable ridge preservation technique, and there is a current need on well-designed and standardized randomized clinical trials in this research field.

## Ethics

Not applicable.

## Studies in humans and animals

Not applicable.

## Informed consent and patient details

Not applicable.

## Declaration of generative AI and AI-assisted technologies in the manuscript preparation process

During the preparation of this work the authors used ChatGPT (OpenAI, San Francisco, California, USA) in order to assist with the revision of English grammar, spelling, and text coherence, without generating any content. After using this tool/service, the authors reviewed and edited the content as needed and take full responsibility for the content of the published article.

## Authors’ contributions

Daniela Meneses-Santos: Contributed to the conception of the work, data acquisition, analysis, and interpretation; drafting and critical review of the manuscript; approval of the final version.

Anderson Carlos Marçal: Contributed to the conception of the work, data acquisition, analysis, and interpretation, drafting and critical review of the manuscript; approval of the final version.

Caio Melo Mesquita: Contributed to the data acquisition, analysis, and interpretation; drafting and critical review of the manuscript; approval of the final version.

Walbert de Andrade Vieira: Contributed to the data acquisition, analysis, and interpretation; drafting and critical review of the manuscript; approval of the final version.

Marcelo Dias Moreira de Assis Costa: Contributed to the conception of the work, data analysis and interpretation; drafting and critical review of the manuscript; approval of the final version.

João Botelho: Contributed to the data analysis and interpretation; drafting and critical review of the manuscript; approval of the final version.

Sigmar de Mello Rode: Contributed to the data analysis and interpretation, drafting and critical review of the manuscript; approval of the final version.

Luiz Renato Paranhos: Contributed to the conception of the work, data acquisition, analysis, and interpretation; drafting and critical review of the manuscript; approval of the final version.

## Funding

This study was financed in part by the Coordenação de Aperfeiçoamento de Pessoal de Nível Superior ‒ Brazil (CAPES) ‒ Finance Code 001. We are also thankful for the support of Conselho Nacional de Desenvolvimento Científico e Tecnológico ‒ Brazil (CNPq) and Fundação de Amparo à Pesquisa do Estado de Minas Gerais (FAPEMIG).

## Conflicts of interest

The authors declare no conflicts of interest.

## Data Availability

All data generated or analyzed during this study are included in this published article and its Supplementary Materials. The datasets used and/or analyzed during the current study are available from the corresponding author on reasonable request.
